# Released Mitochondrial DNA Following Intestinal Ischemia Reperfusion Induces the Inflammatory Response and Gut Barrier Dysfunction

**DOI:** 10.1038/s41598-018-25387-8

**Published:** 2018-05-09

**Authors:** Qiongyuan Hu, Huajian Ren, Jianan Ren, Qinjie Liu, Jie Wu, Xiuwen Wu, Guanwei Li, Gefei Wang, Guosheng Gu, Kun Guo, Zhiwu Hong, Song Liu, Jieshou Li

**Affiliations:** 1 Department of Surgery, Jinling Hospital, Medical School of Nanjing University, Nanjing, China; 2 Jinling college, Nanjing Medical University, Nanjing, China; 3 Department of General Surgery, Nanjing Drum Tower Hospital, the Affiliated Hospital of Nanjing University Medical School, Nanjing, China

**Keywords:** Diagnostic markers, Infection, Sepsis

## Abstract

Ischemia-reperfusion (I/R) injury is a challenging clinical problem, especially injuries involving the gastrointestinal tract. Mitochondrial DNA (mtDNA) is released upon cell death and stress, and can induce the inflammatory response. We aimed to investigate the role of mtDNA in the pathogenesis of intestinal I/R. Intestinal I/R model was established with clamping of the superior mesenteric artery, and IEC-6 cells were incubated under hypoxia/reoxygenation (H/R) conditions to simulate I/R injury. Using *in vitro* models, H/R up-regulated oxidative stress, disrupted mitochondrial activity and the mitochondrial membrane potential, induced apoptosis and elevated the mtDNA levels in the supernatant of intestinal epithelial cells, and the co-culture of mtDNA with human primary dendritic cells significantly elevated TLR9-MyD88 expression and enhanced the production of inflammatory cytokines and chemokines. MtDNA was also released in a mouse model of intestinal I/R and was associated with the increased secretion of inflammatory cytokines and increased gut barrier injury compared with that of the sham group. We concluded that mtDNA contributes to I/R injury and may serve as a biomarker of intestinal I/R. We further suggest that oxidized mtDNA originated from IECs during intestinal I/R exacerbates the acute proinflammatory process by eliciting the production of proinflammatory cytokines and chemokines.

## Introduction

Ischemia-reperfusion (I/R) injury is a clinical problem, especially injuries involving the gastrointestinal tract. The intestine is believed to be the tissue that is most vulnerable to I/R injury^1^. I/R injury to the intestinal epithelium occurs in trauma and shock, especially in infections that may induce hypoperfusion^2^. Ischemia induces tissue disruption because of dysfunction of oxygen delivery, accumulating of toxin and depletion of cellular energy, resulting in cell necrosis. Paradoxically, restoration of the blood supply causes additional cell damage and amplifies the inflammatory response via excess reactive oxygen species (ROS) and nitrogen species, thereby aggravating organ injury and apoptosis^3^.

The gut has long been characterized as the motor of multiple organ dysfunction syndrome (MODS). This characterization may be due to dysregulated crosstalk between the epithelium, immune system, and endogenous microflora of the gut in which the loss of balance between these highly interrelated systems leads to the development of systemic manifestations of disease^4^. Intestinal I/R injury is one of the main triggers that can lead to systemic inflammatory response syndrome and MODS^5^. Although extensive investigation efforts have focused on characterizing the pathogenesis of distant intestinal injury and organ dysfunction, the underlying mechanisms remains the subject of debate, and no effective method exists to prevent or control the process.

A key contributor to the initiating inflammatory response in I/R injury is the activation of endogenous ‘danger signals’ known as damage-associated molecular patterns (DAMPs), which are analogous to pathogen-associated molecular patterns (PAMPs) of infectious pathogens. This sterile inflammatory insult is known to activate innate immunity and propagate organ damage through the recognition of DAMPs^6^. Under various critical conditions, especially after cellular damage and stress, DAMPs are generally released and play an important role in the development various inflammatory diseases^7^. Accumulating evidence has demonstrated an association between the release of DAMPs following I/R and activation of inflammatory response, disruption of the tissue matrix and organ dysfunction^8^.

A recent study has clearly demonstrated that mitochondria are major sources of DAMPs^9^. DNA within mitochondria, called mtDNA, known as DAMPs, is especially crucial for host immune responses and inflammatory activation. MtDNA is resident in the mitochondrial matrix, encased within a double-membrane system composed of the outer and inner mitochondrial membrane^9^. However, because of mitochondrial stress and damage, fragments of mtDNA are released into the cytosol and then into the systemic circulation, leading to systemic inflammation and organ dysfunction^7^. Moreover, an elevated plasma mtDNA level was significantly associated with the occurrence of MODS^10^.

mtDNA can induce inflammatory activation through TLRs, NLRP3, and the cGAS-STING signal pathway^11^. Additionally, an increasing number of studies have revealed that circulating mtDNA functions as DAMPs in traumatic, infectious, and ICU patients and that blood products containing mtDNA might be potential effectors of transfusion-related acute lung injury^12^. Although previous studies have investigated the ability of circulating mtDNA to mediate organ injury following trauma or infections, the involvement of mtDNA, which are closely associated with TLRs in intestinal I/R, has not been examined. Therefore, we hypothesize that intestinal hypoxia and ischemia result in injury to intestinal epithelial cells (IECs) and their mitochondria, which release various DAMPs (mainly mtDNA); the circulating mtDNA initiates SIRS and causes extensive damage to the intestinal mucosal membrane via the circulatory system.

In the present study, we demonstrate that both *in vivo* and vitro I/R models of the mtDNA level was significantly elevated. In addition, the co-culture of mtDNA and plasmacytoid dendritic cell lines (pDCs) showed an increase in inflammatory cytokines secretion. The present study revealed a dominant role for mtDNA in the pathogenesis of intestinal I/R and revealed its potential as a biomarker following I/R injury.

## Results

### Plasma mtDNA increase in IAI patients

**Figure 1:**
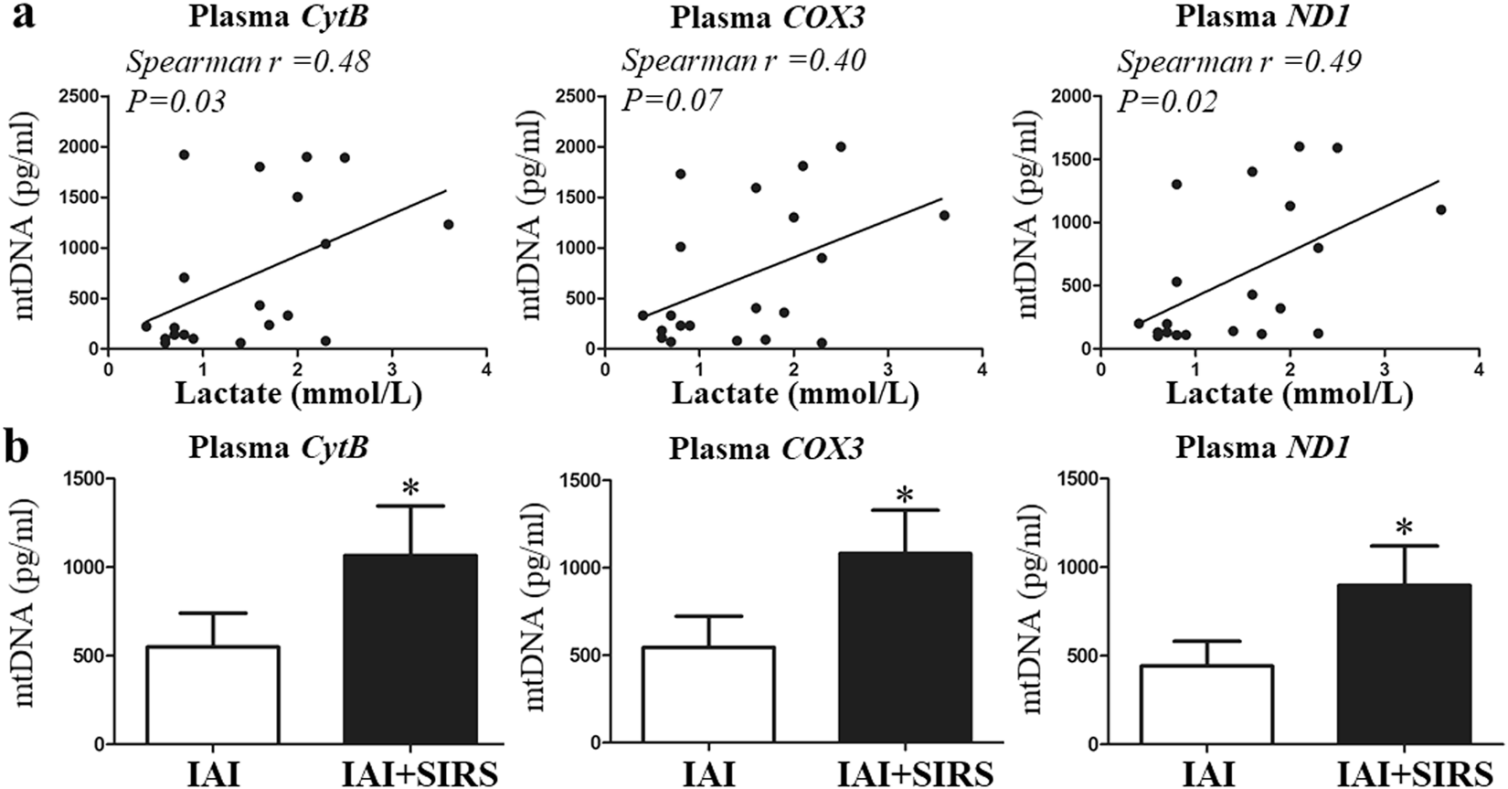
The plasma mtDNA level is increased in IAI patients and is correlated with the lactate level. Three selected sequences were used to detect the mtDNA levels using qPCR. (**a**) Correlations between the baseline mtDNA levels and admission lactate concentration. (**b**) Plasma mtDNA levels between the IAI group and IAI + SRIS group. mtDNA concentrations are presented as means ± SD. *means P < 0.05.

The intestine is the most vulnerable organ to ischemia injury, and intra-abdominal infection (IAI) could cause intestinal I/R to a great extent^4,13^. Therefore, we first clinically explored the relationship between the circulatory mtDNA levels and ischemia injury, and evaluated the role of mtDNA in the inflammatory response (SIRS). Correlation analysis showed that the plasma mtDNA level was correlated with the lactate concentration (Fig. 1a), and the mtDNA level was significantly elevated in IAI patients with SIRS than in those without SIRS (Fig. 1b).

### Oxidative stress, circulating mtDNA, and the inflammatory response increases I/R *in vivo*

**Figure 2:**
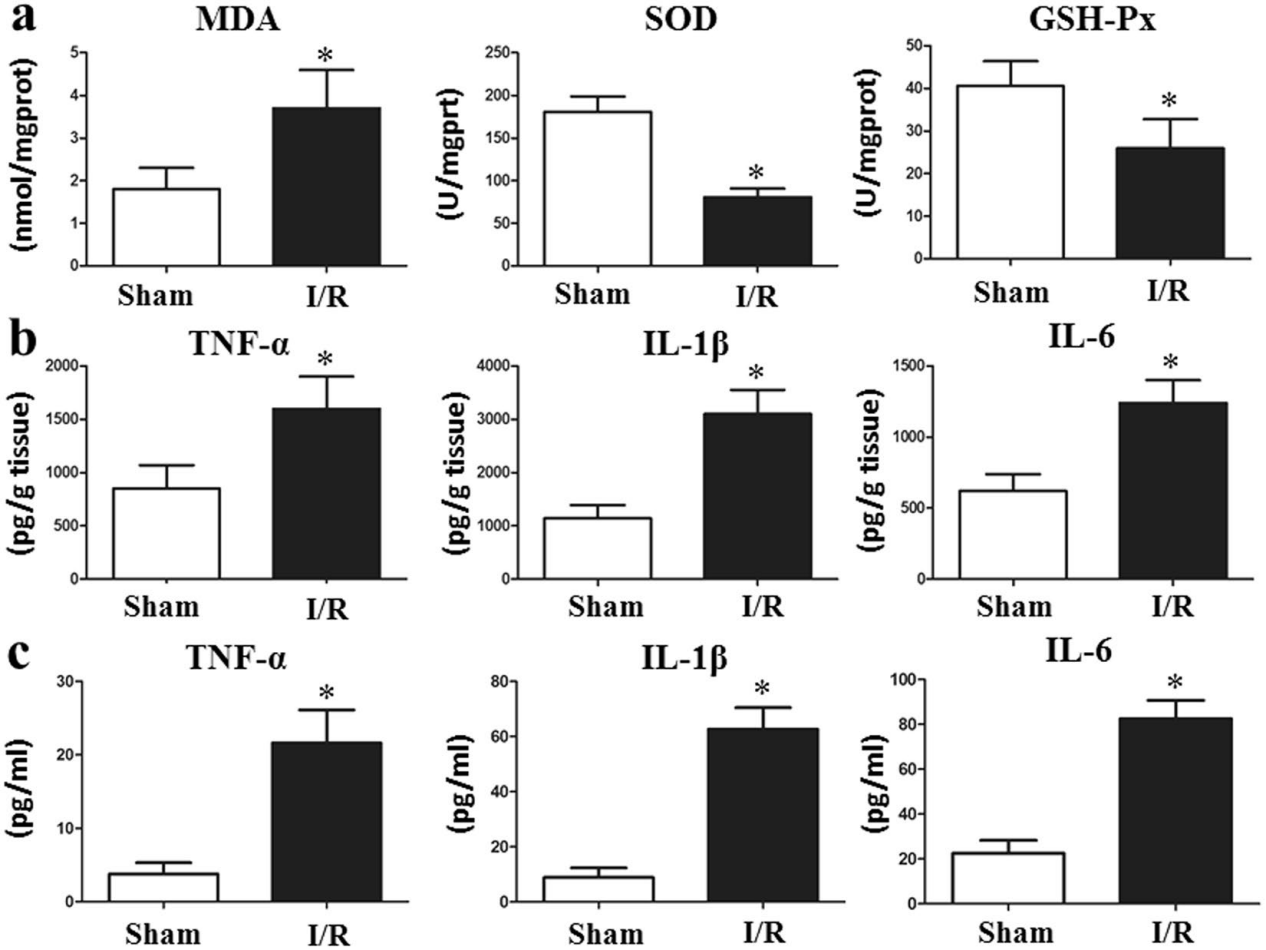
Total intestinal I/R causes oxidative stress and inflammation *in vivo*. (**a**) The levels of SOD and GSH-Px were significantly decreased, and the MDA level was markedly increased in the I/R group compared with that in the sham group. Both the tissue (**b**) and serum (**c**) IL-1β, IL-6 and TNF-α levels production were found to be markedly increased after only two hours of intestinal I/R. The Data were expressed as means ± SD. *p < 0.05 vs the I/R group.

**Figure 3:**
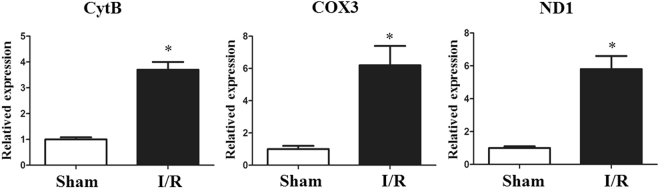
Intestinal I/R increases the mtDNA level in the circulation *in vivo*. Plasma mtDNA levels were significantly elevated in mice undergoing I/R compared with those in the sham group. The data were expressed as means ± SD. *p < 0.05 vs I/R group.

The levels of SOD and GSH-Px were significantly decreased and the MDA level was markedly increased, in the I/R group compared with those in the sham group (Fig. 2a). Some other oxidative indexes were performed in Supplementary Table 2. We next assessed the serum and tissue levels of IL-1β, IL-6, and TNF-α by ELISA in the I/R and sham group. Among the cytokines examined, both the serum and tissue IL-1β, IL-6 and TNF-α production levels were found to be markedly increased after just two hours of intestinal I/R (Fig. 2b,c). In our vivo model, the I/R group displayed a significant increase in mtDNA compared with that in the control group (Fig. 3). Moreover, the increase in inflammatory cytokines was associated with an increase in circulatory mtDNA (data not shown).

### I/R triggers gut injury and intestinal barrier disruption

**Figure 4:**
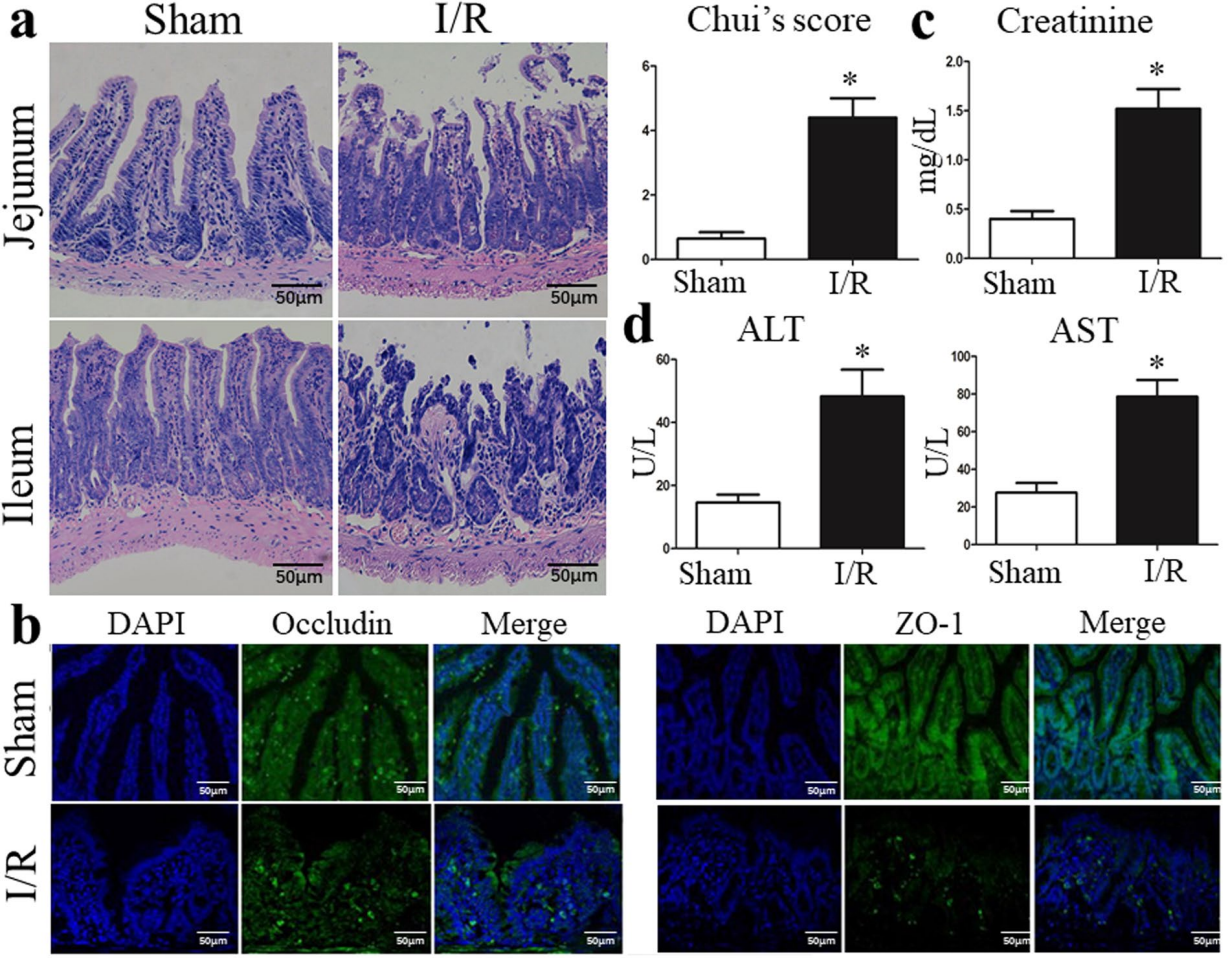
I/R triggers gut injury and intestinal barrier disruption. (**a**) Representative images of intestinal histology (H&E staining; original magnification, ×200) and histopathological scores (Chiu’s score) of the intestine after intestinal I/R. (**b**) Localization of occludin and ZO-1, and DAPI staining within intestinal tissue sections as assessed by immunofluorescence at 6 hours after intestinal I/R. The levels of plasma (**c**) creatinine, (**d**) ALT and AST were also measured by ELISA at 6 hours after I/R. The data were expressed as means ± SD. *p < 0.05 vs the I/R group.

We next sought to confirm that intestinal I/R leads to the injury of IECs and gut barrier *in vivo*. HE staining in the sham group indicated an intact structure of the intestinal mucosa. Conversely, the I/R group was characterized by serious intestinal mucosal injury, short and thin villi, wider villi spacing, necrosis and the collapse of intestinal mucosal epithelial cells, infiltration by inflammatory cells, gap formation beneath the epithelium, lamina propria edema, and capillary hemorrhage (Fig. 4a).

The tight junction (TJ) plays a dominant role in the maintenance of mucosal barrier function. Transmembrane elements, such as occludin and peripheral membrane proteins ZO-1 are major components of the TJ. Immunofluorescence analysis was performed to evaluate the influence of I/R on the morphology of the TJ. As shown in Fig. 4b, the structures of the TJ were intact in the sham group, whereas I/R destroyed the TJ and I/R significantly decreased the expression of TJ proteins compared with that in the sham group by Western blot analysis (Supplementary Fig. 1a). In addition, I/R elevated the serum creatinine (Fig. 4c), ALT and ALT levels (Fig. 4d), indicating hepatic and renal dysfunction following intestinal I/R.

### I/R elevates oxidative stress and mtDNA release ***in vitro***

**Figure 5:**
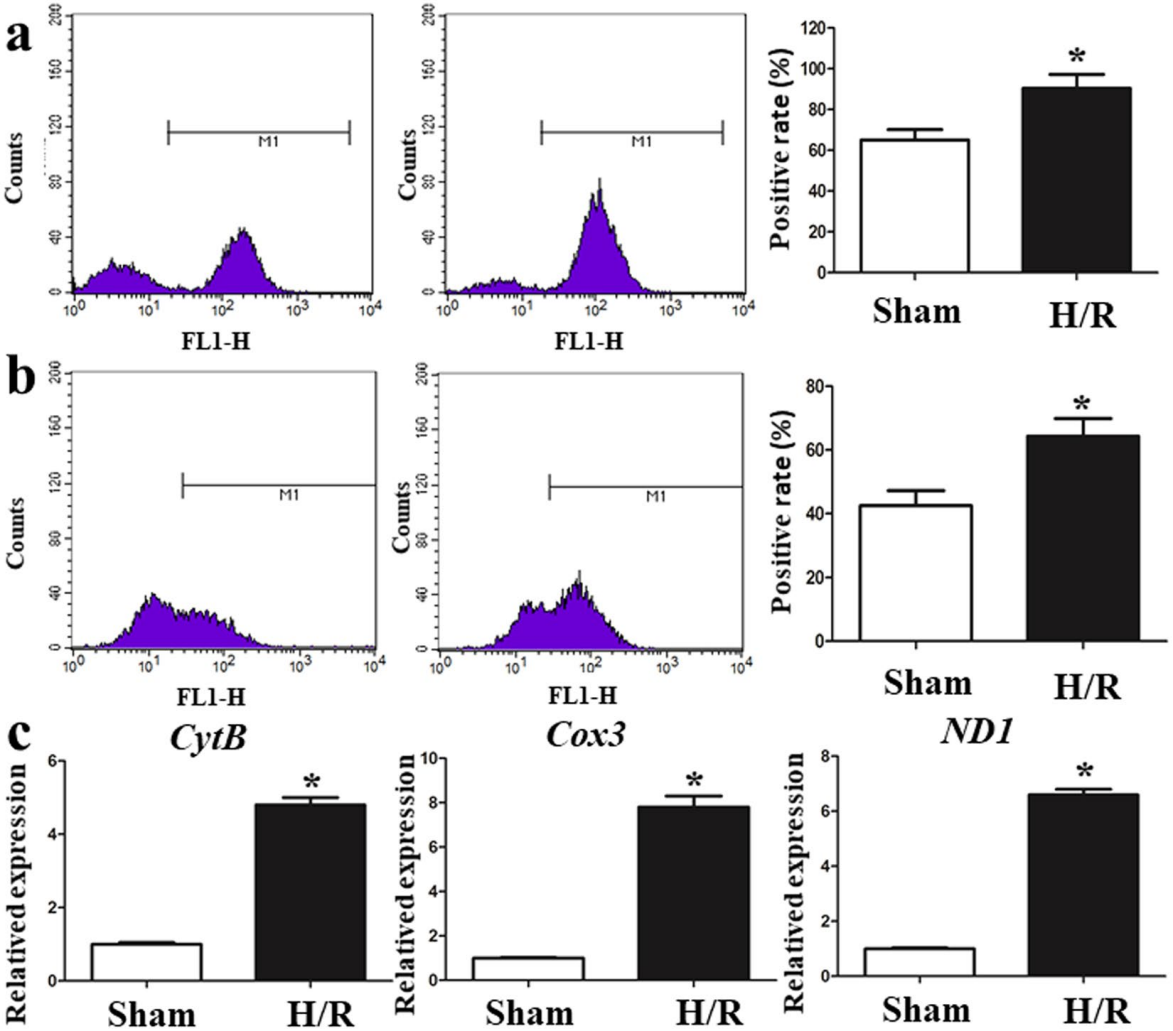
I/R elevates oxidative stress and mtDNA *in vitro*. (**a**) The intracellular ROS levels were increased in H/R-treated IEC-6 cells. (**b**) H/R treatment obviously elevated mitochondrial ROS in IEC-6 cells. (**c**) Cells with H/R exposure showed significantly higher mtDNA levels than normoxic controls. The data are expressed as means ± SD. *P < 0.05 vs the H/R group.

Our previous results demonstrated that oxidative stress was involved in I/R injury *in vivo*. Consistent with this finding, we found that HR of the IEC-6 cell lines significantly increased the end product of lipid peroxidation, MDA, and the level of GSH-Px was obviously decreased (Supplementary Fig. 1b). Intracellular ROS production was measured by flow cytometry during exposure to H/R and the intracellular ROS levels were increased in H/R-treated IEC-6 cells (Fig. 5a). Mitochondrial ROS generation was measured using the MitoSOX Red assay. Figure 5b demonstrates that H/R-treatment obviously elevated mitochondrial ROS in IEC-6 cells. To investigate further the effect of H/R on mtDNA release, 3 selected sequences were used to detect the mtDNA levels. Figure 5c reveals that cells experiencing H/R showed significantly higher mtDNA levels than normoxic controls.

To further confirm the relationships between oxidative stress and the increase in mtDNA, IEC-6 cells were exposed to 500 µM of H_2_O_2_ for 6 h to induced apoptotic cell death. As shown in Supplementary Fig. 2a, H_2_O_2_-induced mitochondrial dysfunction was detected by the decreased mitochondrial membrane potential (ΔΨm) and ATP levels. Moreover, H_2_O_2_ significantly increased IEC-6 cell apoptosis and the mtDNA levels compared with normoxic controls (Supplementary Fig. 2b). These results indicated that increased oxidative stress was significantly associated with an elevation of mtDNA.

### I/R induces cell death

**Figure 6:**
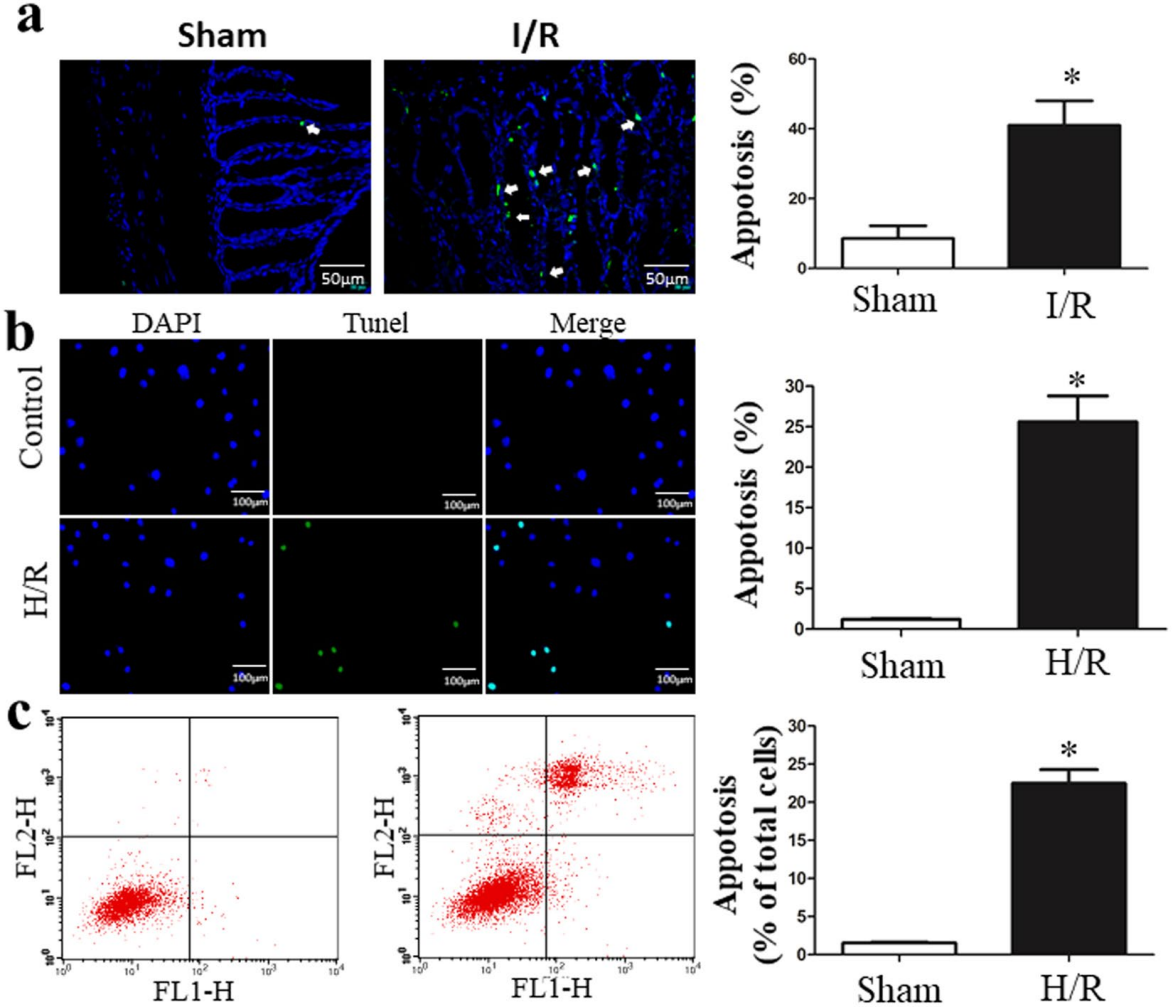
I/R induces cell death. (**a**) Intestinal I/R caused fragmentary or pyknotic-like nuclei, and the chromatin was aggregated in the areas surrounding the nuclei. (**b**) Representative images and the apoptosis index of *in situ* TUNEL assay of IEC-6 cells. TUNEL-positive is green and DAPI is blue. (**c**) AnnexinV/PI analysis with flow cytometry confirmed that H/R-induced cell death. Data are expressed as mean ± SD. *P < 0.05 vs H/R group.

Intestinal epithelial cell apoptosis is considered one of the critical accelerants of gut epithelial integrity disruption^14^. Figure 6a shows that intestinal I/R causes fragmentary or pyknotic-like nuclei, and the chromatin was aggregated in the areas surrounding the nuclei. We also used TUNEL staining and flow cytometry analyses *in vitro* using IEC-6 cells. H/R induced a significant increase in TUNEL-positive apoptotic cells, and the results of the AnnexinV/PI analysis with flow cytometry confirmed that H/R-induced cell death (Fig. 6b,c).

### I/R causes Mitochondrial dysfunction

**Figure 7:**
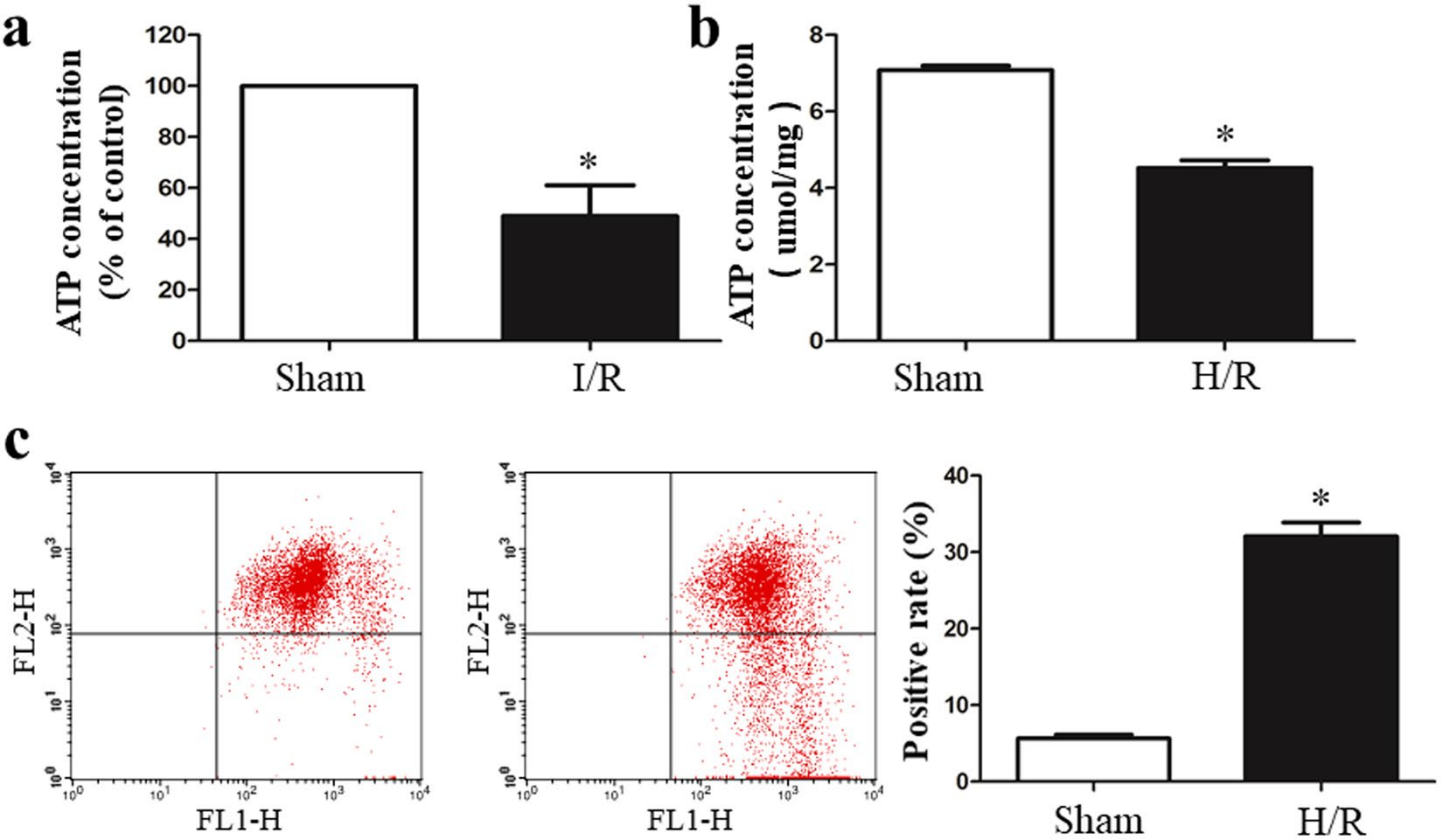
I/R caused mitochondrial dysfunction. The ATP concentration was quantified using a commercially available kit. (**a**) The ATP concentrations were significantly decreased in the I/R group *in vivo*. (**b**) H/R decreased the ATP levels compared with those in the control group. (**c**) The H/R-treated IEC-6 cells showed a decrease in the mitochondrial membrane potential (ΔΨm). The data are expressed as means ± SD. *P < 0.05 vs the sham group or H/R group.

The small intestine is one of the richest organs in terms of the number and density of mitochondria. Many intestinal diseases are associated with the accumulation of damaged mitochondria. The mitochondrial ATP concentrations, an indicator of mitochondrial activity, was quantified. As shown in Fig. 7a,b, the ATP concentration was significantly decreased in the I/R group *in vivo* and *in vitro*. To further investigate the effects of I/R on the mitochondrial function, mitochondrial membrane potential (ΔΨm) was used to detect mitochondrial activity *in vitro* in IEC-6 cells. Figure 7c showed that the IEC-6 cells showed a decrease in ΔΨm After H/R treatment.

### MtDNA induces pDC cell line up-regulation of TLR9 expression and induces an inflammatory response *in vitro*

**Figure 8:**
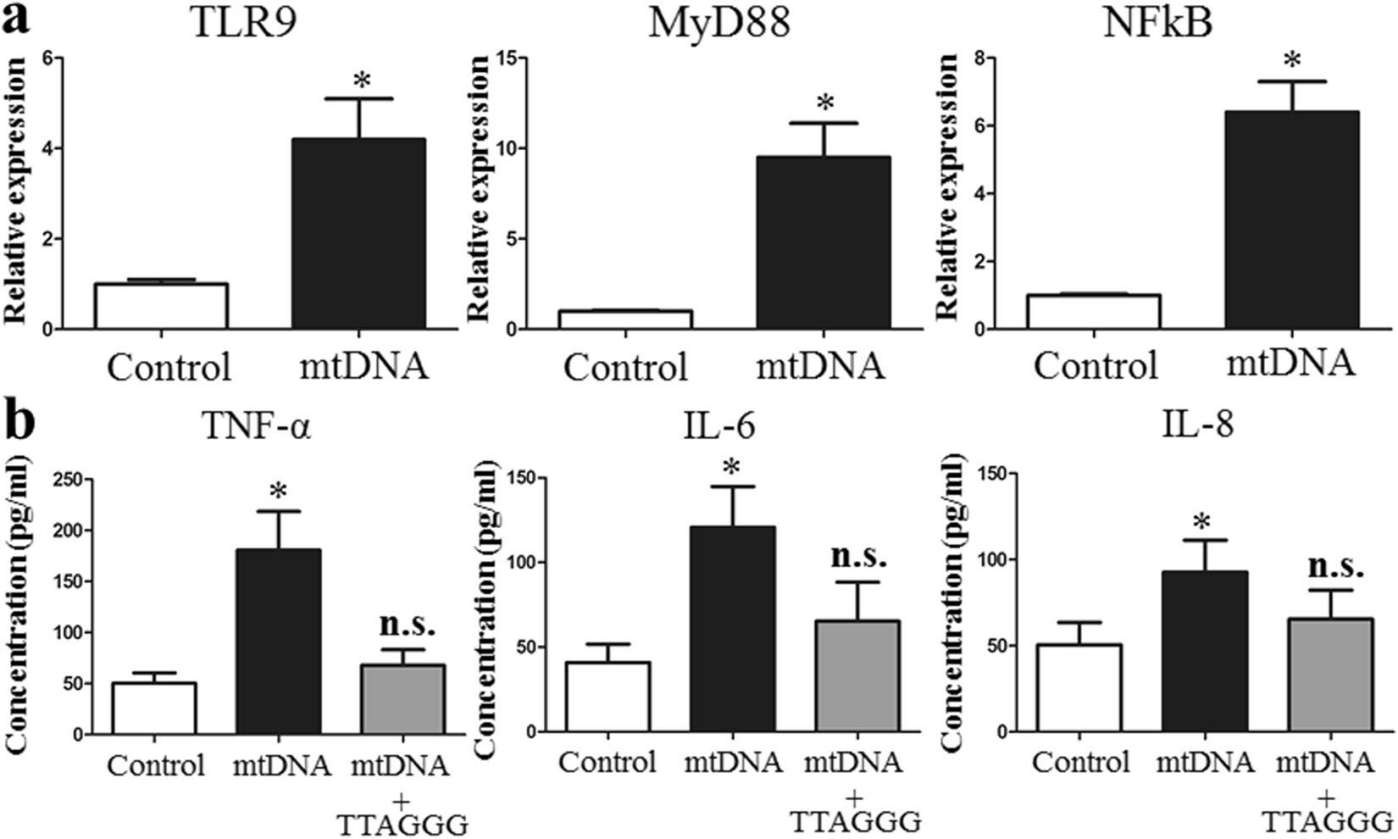
mtDNA induces pDC cell lines to up-regulate TLR9 expression and induced the inflammatory response. (**a**) The expression levels of TLR9, MyD88, and NF-κB was significantly increased with mtDNA co-culture compared with those in the control. (**b**) Freshly isolated primary human pDCs were treated with oxidatively modified mtDNA in the absence or presence of a TLR9 antagonist (TTAGGG). The data are expressed as means ± SD. *P < 0.05 vs the sham group or H/R group.

To further investigate the effect of mtDNA in the inflammatory response, freshly isolated pDCs were treated with oxidized mtDNA purified by H/R treated human IEC-6 cells. The expression levels of TLR9, MyD88, and NF-κB mRNA were significantly increased when pDCs were co-cultured with 10 µg/ml mtDNA (Fig. 8a). Wo also assayed the proinflammatory cytokines, IL-6, IL-8 and TNF-α, in the supernatants of the cell cultures using ELISA. The stimulation of primary pDCs with oxidized mtDNA resulted in the enhanced production of three inflammatory mediators (Fig. 8b). Additionally, the presence of a TLR9 antagonist (TTAGGG) prevented the enhanced IL-6, IL-8 and TNF-α levels in pDCs induced by mtDNA, suggesting that the activation of pDCs was initiated by TLR9-dependent recognition of mtDNA.

## Discussion

The plasma mtDNA concentration is enhanced during sepsis and traumatic injury, and it not only closely correlates with injury severity, but also predicts the risk of post-traumatic systemic inflammatory response syndrome^15^. Our recent study found that circulatory mtDNA was closely associated with the occurrence of sepsis, MODS, and death in patients with IAIs caused by severe abdominal trauma^10^. In addition, a recent study revealed that mtDNA DAMPs in transfusion products significantly contribute to the incidence of ARDS after massive transfusion^12^. Furthermore, circulating mtDNA can activate human neutrophils through TLR9, leading to their migration and degranulation^16^. Because mtDNA can be released from various cell types during infections or under inflammatory conditions, it is closely associated with oxidative stress. In this work we examined the role of oxidized mtDNA in the regulation of inflammation and chemotaxis following intestinal ischemia reperfusion.

mtDNA is particularly vulnerable to oxidative stress because of its special location and structure^17^. mtDNA oxidative injury reduces the synthesis of the respiratory complexes, hinders mitochondrial biogenesis, and leads to mitochondrial dysfunction. In turn, mitochondrial dysfunction enhances ROS production and aggravates damage to the mtDNA that is associated with increased cell death^18^. Consistent with this statement, our present study demonstrated that intestinal I/R up-regulated oxidative stress and intracellular ROS, reduced mitochondrial activity, inhibited ATP generation, decreased the mitochondrial membrane potential, and induced IECs apoptosis. Because of the increased cell death and mitochondrial damage, fragments of mtDNA will be released into the cytosol and then into the systemic circulation. These results suggest that mtDNA may serve as a marker for intestinal I/R injury.

The pathogenesis of intestinal I/R injury was found to be multifactorial and includes oxidative stress, inflammation, and intestinal epithelial barrier disruption, and emerging evidence has revealed that endogenous DAMPs (mtDNA) play a vital role in the initiation of I/R injury and remote organ injury^16,19^. However, none of the studies have illuminated the initiative role for mtDNA in intestinal I/R. In the present study, we demonstrated that mtDNA was elevated by IECs following I/R *in vitro* and in the circulation after intestinal I/R *in vivo*. We further showed that intestinal I/R could contribute to the stressed, damaged or even dying IECs and mitochondrial dysfunction, resulting in elevated mtDNA levels. Importantly, we provided evidence that oxidized mtDNA could trigger a robust inflammatory response of human primary pDCs. These results suggest that mtDNA not only serves as a marker for intestinal I/R injury but may plays an active and initiative role in furthering inflammation and cell death.

We showed that oxidized mtDNA from HR-treated IEC-6 cells could trigger the enhanced production of proinflammatory cytokines and the IL-8 chemokine *in vitro*, revealing that circulatory mtDNA serve as a toxic mediator that participates in the pathogenesis of intestinal I/R injury. It was reported that mtDNA and formyl peptides are released into the circulation following severe trauma, and mtDNA was further increased 24 h after injury. Researchers have also examined whether mtDNA can activate neutrophil migration, and circulating mtDNA was suggested to elicit neutrophil-mediated organ injury^15^. Previous studies have found that besides reactive oxygen species, circulatory DAMPs, as released toxic mediators, are involved in the migration of neutrophils following the late phase of I/R^16^. Based on our result, we hypothesize that mtDNA plays a role in neutrophil activation and in late neutrophil-mediated intestinal I/R injury. Our results found that the expression levels of NF-κB and TNF-α were up-regulated in human pDCs co-cultured with mtDNA. It was demonstrated that stimulation with TNFα can induce either apoptosis or necrosis^20^. These findings suggested that secondary necrotic cell death might be associated with enhanced levels of inflammatory cytokines (TNF-α) induced by mtDNA.

I/R has long been recognized as sterile inflammation, although the situation is more complex when considering intestinal I/R because of the presence of various localized microorganisms such as commensal bacteria and pathogens^13^. Gut barrier dysfunction and bacteria translocation are common features of intestinal I/R injury that enhance intestinal I/R injury. Uncontrolled inflammation could cause gut immunologic barrier dysfunction and damage intestinal tissues. In the present study, intestinal tissue proinflammatory cytokines (IL-1β, IL-6, TNF-α) were significantly elevated. Previous studies have investigated that increased tissue cytokines were mainly related to the infiltration of white blood cells in the late phases of I/R injury, while there was a marked increase in the IL-6 and IL-10 levels just one hour post-reperfusion, during the early phase without neutrophil or monocyte infiltration^21^. Thus, we think that the release of inflammatory cytokines in our experiments was likely related to the activation of resident intestinal macrophages or dendritic cells. Hu *et al*. revealed that mitochondrial DAMPs can trigger apoptosis and necrosis *in vitro* and play a role in the activation of Kupffer cells or dendritic cells to induce an acute inflammatory response^16^. Therefore, based on our results, it is suggested that the released mtDNA caused by intestinal I/R could activate resident immune cells and enhance the production of inflammatory cytokines in the intestinal tract.

Zhang *et al*.^15^ have demonstrated that mtDNA could cause neutrophil-mediated organ injury related to increased TNF-α and IL-6 secretion. Several studies have indicated that the TLR9-MyD88 signaling pathway participates in intestinal I/R injury. Victoni *et al*.^13^ investigated that intestinal inflammation and injury are attenuated in the absence of MyD88 and are associated with reduced neutrophil recruitment and diminished proinflammatory cytokine production. A previous study also demonstrated that oxidized mtDNA by tail vein injection could stimulate the inflammatory mediator and chemokine levels^21^. In the present study, the presence of a TLR9 antagonist prevented enhanced inflammatory production from pDCs induced by oxidative mtDNA. Based on previous evidence and our results, we suggest that intestinal I/R injury causes a significantly increased release of mtDNA originated mainly from IECs, thus leading to local and remote inflammation through inducing the TLR9/MyD88 signal in immune cells. This may include neutrophils, intestinal macrophages, dendritic cells, and infiltrating monocytes, which cause inflammation by secreting proinflammatory cytokines. In this study, we first explored whether mtDNA might may play an active and initiative role in furthering inflammation and cell death.

Ischemia reperfusion can elicit an inflammatory response mediated by TLRs, which are germline-encoded, pattern-recognition receptors that are highly conserved and broadly expressed in tissues^13^. Previous evidence has suggested that massive intestinal barrier disruption, including damage to the microvasculature, may allow the escape of several TLR agonists produced by intestinal commensals or direct translocation of intestinal microbes into the circulation, leading to systemic inflammation and organ injury^22,23^. In general, gut barrier damage caused by intestinal I/R is localized, and our study indicates that released mtDNA from the IECs plays an initial role in amplifying the local inflammation conditions, leading to the early phase of intestinal mucosa injury and causing the damage of vascular endothelium and organs. Previous evidence has revealed that circulatory mtDNA was suggested to initiate cardiac, pulmonary and hepatic dysfunction, as well as the inflammatory response^12,16,24–26^. Furthermore, a recent study has demonstrated that mtDNA was involved in the stimulation of chemokine expression and activation of vascular inflammation^27^. These lines of evidence suggest that mtDNA combined with PAMPs may contribute to SIRS and organ injury. When the clinical source control prevents the PAMPs from damaging organ function, DAMPs (mtDNA) may also play an important role in sustained injury. Therefore, in the clinic, when patients with intestinal I/R develop bacteremia, antimicrobial chemotherapy may not be sufficient, and physicians should focus on mtDNA-related inflammation and organ injury. Further studies are needed to clarify the potential mechanism of the role of PAMPs and DAMPs in intestinal I/R.

In conclusion, the present study demonstrates convincingly, for the first time, that mtDNA contributes to gut I/R injury and may serve as a biomarker of intestinal I/R. Furthermore, our data provide evidence that oxidative mtDNA has great potential to activate human pDCs and initiate the inflammatory response. Our results suggest that oxidized mtDNA originates from stressed, damaged, or even dying IECs during intestinal I/R and may exacerbate the acute proinflammatory process by eliciting the production of proinflammatory cytokines and chemokines from TLR-9-expressing cells. This study identifies a novel marker for intestinal I/R and potentially provides additional targets to ameliorate the sterile inflammatory response associated with ischemia and reperfusion injury.

## Materials and Methods

### Ethics Statement

The present study was approved by the Ethics Committee of Jinling Hospital, Nanjing. Written informed consent was obtained from all enrolled patients/their families and healthy adult volunteers before the study-related procedure was begun.

### Animals and operative procedure

Male C57BL/6 mice (aged 8 weeks) were obtained from the Model Animals Research Center of Nanjing University. Anesthetic management and experimental intestinal I/R establishment were performed as previously described^28^. Briefly, all mice were anesthetized with pentobarbital by intraperitoneal injection. After midline laparotomy, the superior mesenteric artery (SMA) of I/R mice was identified and occluded using an atraumatic microvascular clamp for 30 min; the clamp was then removed to allow for reperfusion. In sham mice, the SMA was isolated but not occluded. During the study period, the animal’s temperature was maintained at approximately 36 °C using a heating pad. Animal experiments were performed in accordance with the Guide for the Care and Use of Laboratory Animals of the Institute for Laboratory Animal Research, and approval to conduct the study was obtained from the Animal Investigation Ethics Committee of Jinling Hospital. The methods were carried out according to the approved guidelines.

### Patients

The present study enrolled 20 intra-abdominal infection patients in the surgical intensive unit (SICU) at Jining Hospital from December 2016 to March 2017. The Definitions of IAIs and systemic inflammatory response syndrome (SIRS) were described as previously^10^. Patient database information in our hospital is either automatically recorded by specialized software (Nanjing Haitai Medical Information System) or is recorded manually by physicians. All methods relating to humans were performed in accordance with the relevant guidelines and regulations in our study.

### Ischemia/reperfusion (I/R) *in vitro* models

A well-established hypoxia/reoxygenation (H/R) model was employed using IEC-6 cells to mimic I/R *in vivo*^1^. IEC-6 cells were cultured in serum-free medium and were maintained at 37 °C in a humidified atmosphere. To establish hypoxic conditions, cells were incubated in a microaerophilic system with 5% CO_2_ and 1% O_2_ balanced with 94%N_2_ for 12 hours. The cells were then transferred to normal conditions to achieve reoxygenation.

### Histopathological Assessment of Intestines

After reperfusion, 1 cm of the small intestine was fixed in 10% formalin and was embedded in paraffin, and 4-*μ*m sections were stained with hematoxylin and eosin for light microscopy. Histological scoring of intestinal tissue injury was assessed according to the method described by Chiu *et al*.^29^ with the following modifications: score 0, no damage; score 1, subepithelial space at the villous tip; score 2, loss of mucosal lining of the villous tip; score 3, loss of less than half of the villous structure; score 4, loss of more than half of the villous structure; and score 5, transmural necrosis. The sections were evaluated blindly.

### Immunofluorescence assessment of the intestinal barrier

Immunofluorescence was used to determine the localization of occludin and, zona occludens-1 (ZO-1) proteins. The intestinal section was washed with PBS, mounted in embedding medium, and stored at −80 °C until use. Frozen sections were cut at 10 μm and were mounted on the slides. The polyclonal antibodies against occludin and, ZO-1 (Abcam, Cambridge, UK) were incubated according to the manufacturer’s instructions. The sections were probed with their respective FITC-conjugated secondary IgG antibodies. The nuclei were counterstained with DAPI (4, 6-diamidino-2-phenylindole). Slides incubated without any primary antibody were using as negative controls. Confocal analysis was performed with a confocal scanning microscope (Leica Microsystems, Heidelberg GmbH, Mannheim, Germany).

### Preparation of mtDNA

Mitochondria were isolated from H/R-treated human intestinal epithelial cells (IEC-6 cell lines) using a mitochondrial isolation kit (BioVision) following the manufacturer’s instructions. mtDNA was purified from mitochondrial pellets using DNeasy blood and sodium chloride. The mtDNA concentrations and purity were analyzed using the NanoDrop 2000 system (Thermo Fisher Scientific, Waltham, MA, USA), and the quality of the mtDNA samples was checked. In addition, to further exclude any significant nDNA contamination and to ensure the purity of the mtDNA, q-PCR was conducted. The nDNA content was less than 0.1% in the isolated mtDNA samples.

### ATP content determination

The adenosine-5′ triphosphate (ATP) content was determined as described previously (Matsui *et al*., 2007) using the ATP Determination Kit (Beyotime). Briefly, the cells (106 cells/ml) or intestinal tissues (1:10 w/v) were lysed and centrifuged at 10,000 g for 15 min. The supernatants (10 ml) were mixed with the reaction buffer (100 ml) containing 1 mM dithiothreitol, 0.5 mM luciferin, and 12.5 mg/ml of luciferase. The luminance (RLU) of the mixtures was measured using a Varioskan Flash (Thermo Scientific) microplate reader. An ATP standard curve was also constructed for calculation, and the ATP levels were expressed as the percentage of the total level that was observed in the control groups.

### Apoptosis detection

Apoptosis in the intestinal sections and IEC-6 cell lines were identified based on the TUNEL staining method using a commercial kit (Roche Diagnostics Corp., Indianapolis, IN, USA) according to the manufacturer’s protocol. The apoptotic index was measured by the percentage of TUNEL-positive cells as previously described. Apoptosis was also examined using the FITC-labeled AnnexinV/propidium iodide (PI) Apoptosis Detection kit (BD Biosciences, Franklin Lakes, NJ) following the manufacturer’s instructions. Briefly, after H/R-treatment, the IEC-6 cells were suspended in 200 ml of 1 × binding buffer (1 × 10^6^ cells/ml). The cells were then incubated with AnnexinV (1:20) for 3 min followed by PI for 15 min in the dark, at room temperature. The cells were subjected to flow cytometry using the a BD FACS Calibur system, and the data were analyzed using FlowJo software.

### Detection of oxidative stress

Oxidative stress in the intestinal tissues and IEC-6 cells was measured using MDA, SOD, and GSH-Px (Nanjing Jiancheng, China) according to the manufacturer’s recommendations. The fluorescent probes DCFH-DA and JC-1 (Sigma) were used to measure the intracellular accumulation of ROS. The two cell lines were plated in 6-well plates at the density of 5 × 104 cells/mL and were treated with H/R. The cells were harvested and then re-suspended in 10.0 μM DCFH-DA and 500 μl of JC-1 solution at 37 °C for 30 min, followed by detection by flow cytometry (Becton-Dickinson, USA).

### Isolation and vitro stimulation of primary pDCs

Peripheral blood mononuclear cells (PBMCs) were isolated from heparinized leukocyte-enriched buffy coats by standard Ficoll-Paque (GE Healthcare) density gradient centrifugation. Untouched human pDCs were purified from PBMCs by negative selection using an immunomagnetic cell separation kit (Miltenyi Biotec, Bergish Gladbach, Germany), according to the manufacturer’s instructions and the purity was confirmed by flow cytometry. For stimulation, pDCs were exposed for 24 h to 10 μg of mtDNA prepared from H/R treated IEC-6 cells, and pDCs were preincubated for 1 h with TLR9 inhibitory ODN TTAGGG oligonucleotide and then was cotreated with 10 μg of mtDNA for 24 h.

### Quantitative real-time PCR

Circulating DNA was isolated from 200 μl of plasma using the QIAamp DNA Blood Mini Kit (Qiagen, Valencia, CA), according to the manufacturer’s protocol. Quantitative real-time polymerase chain reaction (qPCR) and the manufacturer’s protocol were applied to quantify selected *200-bp sequences corresponding to the *CytB*; *COX3*; *ND1* mitochondrial genomic regions as described previously. The following primers were used to determine mRNA expression levels: TLR9, MyD88, NF-κB and glyceraldehyde phosphate dehydrogenase (GAPDH). The primers for qPCR analyses of the relevant sequences are listed in Supplementary Table 1. Briefly, after total RNA was extracted from intestinal mucosa tissues using TRIZOL reagent (Life Technologies Inc., Carlsbad, CA, USA), the oligo (dT)-primed complementary DNA was used for reverse transcription of the purified RNA. The transcript amounts of the genes of interest were measured by quantitative PCR using SYBR Green detection (Applied Biosystems, Carlsbad, CA, USA). The expression levels of each gene were expressed as a relative value to GAPDH and then were normalized to the mean value of the control group. Blood was collected, and plasma mtDNA was detected for all the IAI patients. Cell free plasma samples (200 µL each) were was aspirated and used for DNA extraction using a commercial kit according to the manufacturer’s instructions. The generation of a plasma mtDNA standard curve was accomplished using purified mtDNA, which was extracted from IEC-6 cells and quantified using the absorbance at 260 nm.

### Western blotting analysis

Proteins from the tissues were separated by SDS-PAGE and were transferred to PVDF membranes. Western blotting was performed using antibodies against occludin and ZO-1 (Abcam, Cambridge, UK). Protein quantification was measured in optical density units using Image Lab software (Bio-Rad, CA, USA) and was normalized to the corresponding sample expression of β-actin. The methods for the determination were described previously^14^.

### Biochemical assay

The levels of serum and tissue tumor necrosis factor (TNF-α), IL-1β, IL-6 and IL-8 in mice and cell supernatants were detected using an enzyme-linked immunosorbent assay (ELISA) kit (R&D System). The contents of ALT, AST and creatinine were (Nanjing Jiancheng, China) were measured using commercial kits according to the manufacturer’s instructions.

### Statistical analysis

Data is presented as mean ± SEM and were compared by one way analysis of variance with the nonparametric Mann-Whitney test used to determine differences between groups. Correlation analysis was used to explore plasma mtDNA and lactate in enrolled patients. P value < 0.05 was considered statistically significant.

## Electronic supplementary material

Supplementary information
