# Author Correction: Released mitochondrial DNA following intestinal ischemia reperfusion induces the inflammatory response and gut barrier dysfunction

**DOI:** 10.1038/s41598-022-06686-7

**Published:** 2022-02-09

**Authors:** Qiongyuan Hu, Huajian Ren, Jianan Ren, Qinjie Liu, Jie Wu, Xiuwen Wu, Guanwei Li, Gefei Wang, Guosheng Gu, Kun Guo, Zhiwu Hong, Song Liu, Jieshou Li

**Affiliations:** 1 Department of Surgery, Jinling Hospital, Medical School of Nanjing University, Nanjing, China; 2 Jinling College, Nanjing Medical University, Nanjing, China; 3 Department of General Surgery, Nanjing Drum Tower Hospital, the Affiliated Hospital of Nanjing University Medical School, Nanjing, China

Correction to: *Scientific reports* 10.1038/s41598-018-25387-8, published online 09 May 2018

This Article contains an error in Figure 4, where the images in panel (a) for the Sham group, Jejunuum and the I/R group, Ileum are incorrect.

The correct Figure 4 and accompanying legend appear below.

**Figure 4:**
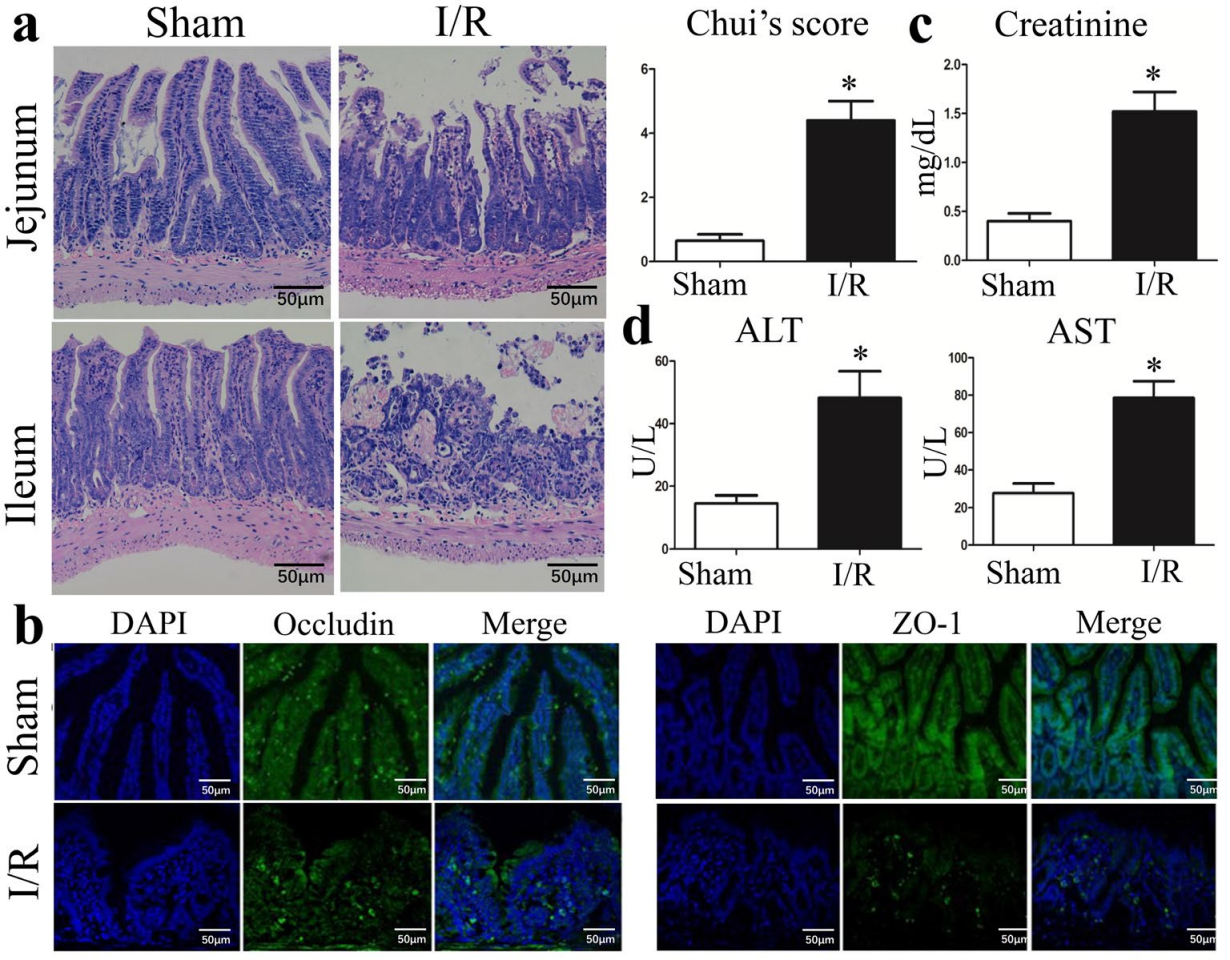
I/R triggers gut injury and intestinal barrier disruption. (**a**) Representative images of intestinal histology (H&E staining; original magnification, 200) and histopathological scores (Chiu’s score) of the intestine after intestinal I/R. (**b**) Localization of occludin and ZO-1, and DAPI staining within intestinal tissue sections as assessed by immunofluorescence at 6 h after intestinal I/R. The levels of plasma (**c**) creatinine, (**d**) ALT and AST were also measured by ELISA at 6 h after I/R. The data were expressed as means ± SD. *p < 0.05 vs the I/R group.

