# Supplementary material for: Released Mitochondrial DNA Following Intestinal Ischemia Reperfusion Induces the Inflammatory Response and Gut Barrier Dysfunction
Source: Sci Rep. 2018 May 9;8:7350. doi: 10.1038/s41598-018-25387-8 (PMC5943336; doi:10.1038/s41598-018-25387-8)
Supplement: Supplementary file 1 — Supplementary information [file 41598_2018_25387_MOESM1_ESM.pdf]

Supplementary information

Title

Released Mitochondrial DNA Following Intestinal Ischemia Reperfusion Induces the Inflammatory Response and Gut Barrier Dysfunction

Qiongyuan Hu[1], MD; Huajian Ren[1, 2], MD; Jianan Ren[1*], MD, FACS; Qinjie Liu[1, 2], MD; Jie Wu[1], MD; Xiuwen Wu[1*], MD; Guanwei Li[1], MD; Gefei Wang[1], MD; Guosheng Gu[1], MD; Kun Guo[1], MD; Zhiwu Hong[1], MD; Song Liu[3], MD; Jieshou Li[1], MD

1Department of Surgery, Jinling Hospital, Medical School of Nanjing University,

2Jinling college, Nanjing Medical University

3Department of General Surgery, Nanjing Drum Tower Hospital, the Affiliated Hospital of Nanjing University Medical School

Table S1. Primers used for quantitative PCR analysis

Genes	Primers sequence
Human cytochrome B	F: 5'- ATGACCCCAATACGCAAAAT -3'
	R: 5'- ATGACCCCAATACGCAAAAT -3'
Human Cox3	F: 5'- ATGACCCACCAATCACATGC -3'
	R: 5'- ATCACATGGCTAGGCCGGAG -3'
Human ND1	F: 5'- ATACCCATGGCCAACCTCCT -3'
	R: 5'- GGGCCTTTGCGTAGTTGTAT -3'
Mouse cytochrome B	F: 5'- TGAGGGGGCTTCTCAGTAGA -3'
	R: 5'- CTGTTTCGTGGAGGAAGAGG -3'
Mouse Cox3	F: 5'- CGTGAAGGAAACTACCCAGG -3'
	R: 5'- CGCTCAGAAGAATCCTGCAA -3'
Mouse ND1	F: 5'- GGATCCGAGCATCTTATCCA -3'
	R: 5'- GGTGGTACTCCCTCTGTAAA -3'
TLR 9	F: 5'- CCTGGCACACAATGACATTCA -3'
	R: 5'- TAAAGGTCCTCCTCGTCCCA -3'
MyD88	F: 5'- GAGATCCGCGAGTTTGAGAC -3'
	R: 5'- TTGTCTGTGGGACACTGCTC -3'
NFκB	F: 5'- GAGGACTTGCTGAGGTTGG -3'
	R: 5'- TGGGGTGGTTGATAAGGAGTG -3'
GAPDH	F: 5'- GGCACAGTCAAGGCTGAGAATG -3'
	R: 5'- ATGGTGGTGAAGACGCCAGTA -3'

Supplementary Table 2. Oxidative and antioxidative parameters in sham and I/R group.

**Parameters**	**Sham**	**I/R**	**P value**
H_2O_2 (mmol/l)	17.5±5.1	25.7±6.5	0.029
$^{\bullet}OH$ (U/mggrot)	236.5±75.1	321.9±66.6	0.047
GSH (mg/gprot)	3.9±0.8	2.2±1.1	0.012
CAT (U/mggrot)	21.1±6.5	13.6±2.1	0.035

Values are mean ±SD. hydrogen peroxide (H_2O_2); hydroxyl radical ($^{\bullet}OH$); glutathione (GSH); catalase (CAT).

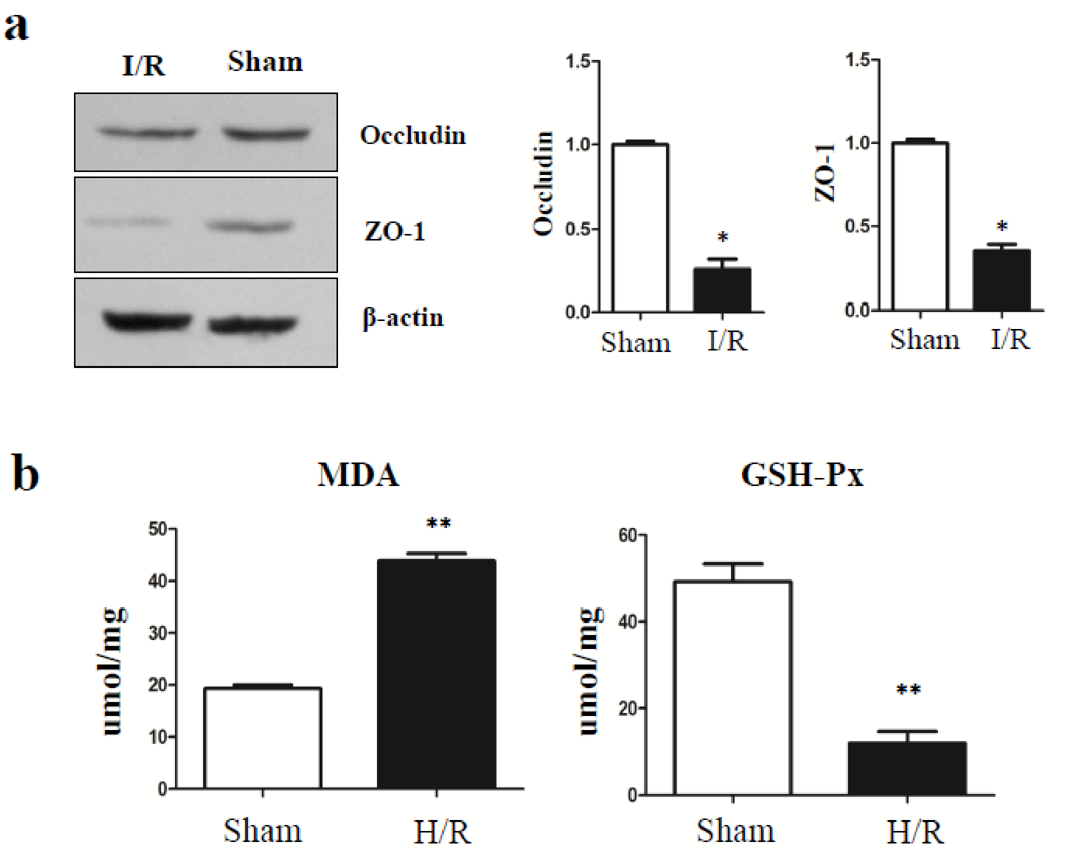

Supplementary Fig.S 1 Ischemia reperfusion significantly decreased the expression of tight junction proteins and elevated oxidative stress in vitro. (a) Occludin and ZO-1 expression were determined by western blot. (b) H/R of the IEC-6 cell lines significantly increased MDA, and the level of GSH-Px was obviously decreased. *$p < 0.05$, **$p < 0.01$ when compared with sham group.

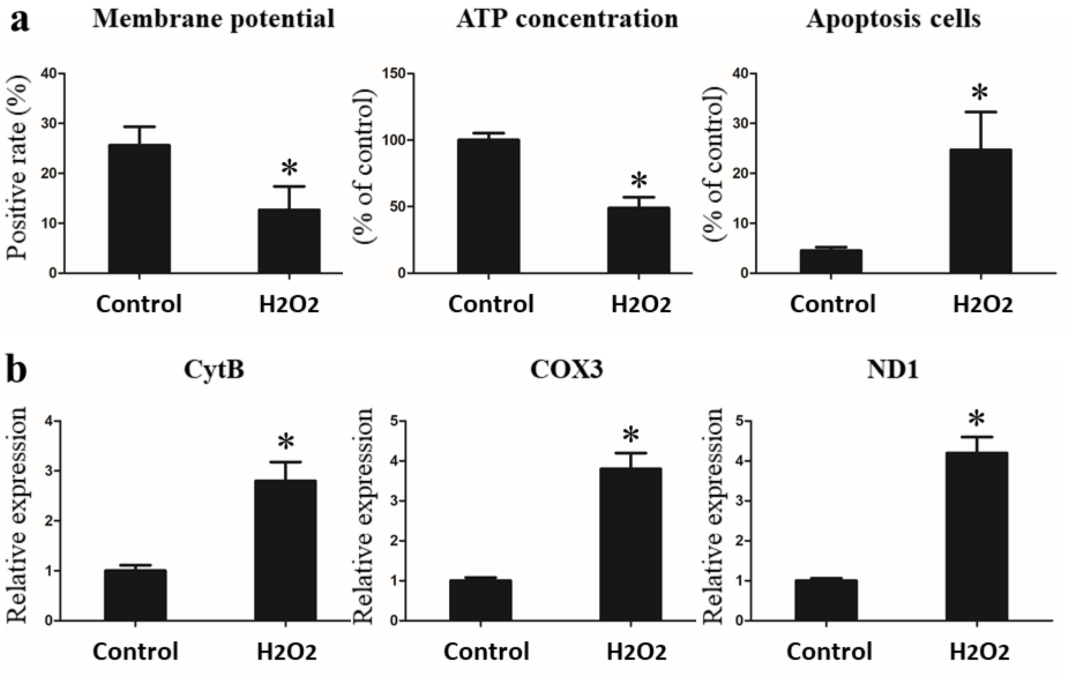

Supplementary Fig.S 2 H_2O_2-induced mitochondrial dysfunction, apoptosis, and mtDNA release. (a) mitochondrial membrane potential, ATP content, and cell apoptosis were quantified after H_2O_2 treatment. (b) detection of mtDNA levels in supernatant of H2O2-treated IEC-6 cells. *P<0.05 vs control.

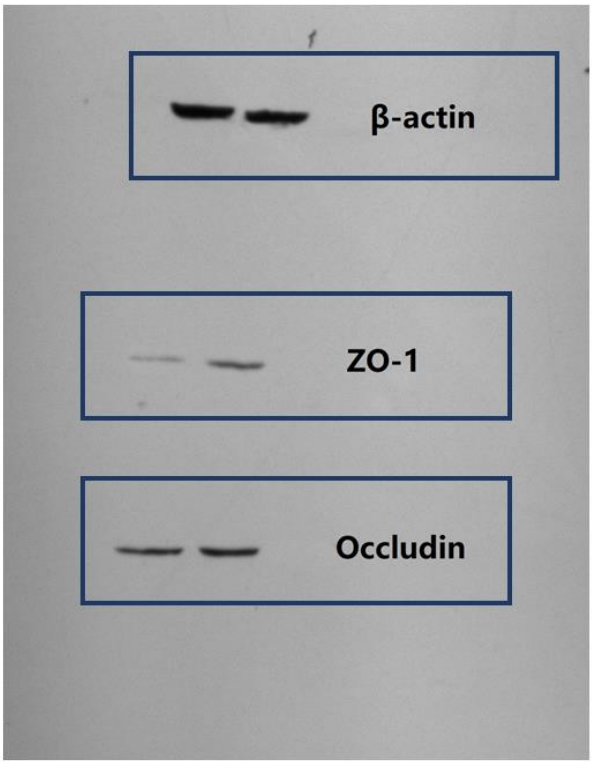

Supplementary Fig.S 3 Unprocessed original scans for the bolts in Figure S1a.
